# *Legionella* Diversity and Spatiotemporal Variation in the Occurrence of Opportunistic Pathogens within a Large Building Water System

**DOI:** 10.3390/pathogens9070567

**Published:** 2020-07-13

**Authors:** Helen Y. Buse, Brian J. Morris, Vicente Gomez-Alvarez, Jeffrey G. Szabo, John S. Hall

**Affiliations:** 1 Homeland Security and Materials Management Division, Center for Environmental Solutions & Emergency Response (CESER), Office of Research and Development (ORD), US Environmental Protection Agency (USEPA), Cincinnati, OH 45268, USA; Szabo.Jeff@epa.gov (J.G.S.); Hall.John@epa.gov (J.S.H.); 2 Pegasus Technical Services, Inc c/o US EPA, Cincinnati, OH 45268, USA; morris.brian@epa.gov; 3 Water Infrastructure Division, Center for Environmental Solutions & Emergency Response (CESER), US Environmental Protection Agency (USEPA), Office of Research and Development (ORD), Cincinnati, OH 45268, USA; Gomez-Alvarez.Vicente@epa.gov

**Keywords:** potable water, first draw, second draw, biofilm, whole genome sequencing, environmental monitoring, premise plumbing systems

## Abstract

Understanding *Legionella* survival mechanisms within building water systems (BWSs) is challenging due to varying engineering, operational, and water quality characteristics unique to each system. This study aimed to evaluate *Legionella*, mycobacteria, and free-living amoebae occurrence within a BWS over 18–28 months at six locations differing in plumbing material and potable water age, quality, and usage. A total of 114 bulk water and 57 biofilm samples were analyzed. *Legionella* culturability fluctuated seasonally with most culture-positive samples being collected during the winter compared to the spring, summer, and fall months. Positive and negative correlations between *Legionella* and *L.* *pneumophila* occurrence and other physiochemical and microbial water quality parameters varied between location and sample types. Whole genome sequencing of 19 presumptive *Legionella* isolates, from four locations across three time points, identified nine isolates as *L. pneumophila* serogroup (sg) 1 sequence-type (ST) 1; three as *L. pneumophila* sg5 ST1950 and ST2037; six as *L. feeleii*; and one as *Ochrobactrum*. Results showed the presence of a diverse *Legionella* population with consistent and sporadic occurrence at four and two locations, respectively. Viewed collectively with similar studies, this information will enable a better understanding of the engineering, operational, and water quality parameters supporting *Legionella* growth within BWSs.

## 1. Introduction

Various *Legionella* species, such as *L. anisa*, *L. feeleii*, *L. longbeachae*, *L. pneumophila*, and *L. micdadei*, can cause legionellosis, which are bacterial infections resulting in either a mild flu-like illness (Pontiac Fever) or a potentially fatal form of pneumonia (Legionnaires’ Disease (LD)) [1]. These infections are primarily caused by the inhalation of *Legionella*-contaminated aerosols generated from engineered water systems [2,3]. Of the 74 drinking water-associated outbreaks reported in the US between 2011 and 2014, *Legionella* was responsible for 61% of those outbreaks causing 17% (241/1437) of the illness cases, 88% (200/226) of hospitalizations, and 100% of the outbreak deaths (27/27) [4,5]. *Legionella* presence in the building water systems (BWSs) was cited as the main deficiency leading to those outbreaks underscoring the need to control and prevent *Legionella* growth within these BWSs.

Over the past 40 years, *Legionella* occurrence in drinking water distribution systems (DWDSs) has been well-studied since the first speculation that exposure to aerosols, derived from contaminated water chillers, caused the deadly 1976 American Legion convention outbreak in Philadelphia [6]. *Legionella* has been shown to occur at low levels in drinking water treatment plants and distribution systems and at higher levels in both cold and hot water within BWSs [7,8,9]. *Legionella* occurrence has been documented in both antiquated (> 100 years old) and newly constructed buildings [10,11]. Moreover, numerous studies have reported stable colonization of, and continuing infections caused by, *L. pneumophila* within BWSs over a ≥ 15 year period, despite repeated cycles of shock chlorination, superheating and thermal inactivation, and flushing [12,13,14,15,16]. 

*Legionella* occurrence in diverse environments and conditions (e.g., in sea-, fresh-, rain-, and treated water; in soil; at temperatures between 4 and 60 °C and a pH range of 2.7–8.3) [1] suggests the presence of heterogeneous survival mechanisms acquired through effective evolutionary processes. Indeed, *L. pneumophila* is genetically well-equipped due to their (1) high recombination rate and DNA exchange among strains and different *Legionella* species [17] and (2) large repertoire of effector proteins allowing exploitation of conserved cellular pathways in various eukaryotic hosts [18,19].

Due to their stringent nutrient requirements, it is hypothesized that *Legionella* growth within DWDSs is largely dependent on their parasitization of drinking water-associated eukaryotic hosts, specifically free-living amoebae (FLA). FLA, such as *Acanthamoeba* spp. and *Vermamoeba vermiformis*, are problematic in drinking waters systems as they can cause diseases like keratitis and can also amplify other human pathogens such as *L. pneumophila* and mycobacteria, including *Mycobacterium intracellulare* [20,21]. *M. intracellulare*, a member of the non-tuberculous mycobacteria (NTM) group, is a significant cause of pulmonary NTM infections [22] and has been isolated in higher frequencies and concentrations in both bulk water and biofilms from drinking water distribution systems compared to *Mycobacterium avium* [23].

Numerous studies have demonstrated extracellular growth of *L. pneumophila* in drinking water. Specifically, four different types of non-*Legionella* drinking water bacteria [24,25]; heat-inactivated cooling tower biofilms, *Escherichia coli*, and *Pseudomonas putida* [26]; extracellular cyanobacterial components [27]; and filtered sterilized drinking water [28] were capable of supporting *L. pneumophila* growth, with the latter also supporting their colonization and growth within biofilms for prolonged periods. Collectively, *Legionella* associations with, and dependencies on, other microbes can explain their vast environmental distribution, ability to survive in DWDSs, and human pathogenicity, especially in engineered environments where confined, close proximity to humans have increased disease risk from this pathogen.

Thus, current approaches to *Legionella* exposure prevention, e.g., through environmental monitoring [29] and/or implementation of building water management plans [30,31], require a thorough understanding of their persistence and transmission mechanisms in premise plumbing systems. In this study, to better understand BWS conditions supporting *Legionella* survival, microbial water quality parameters, such as the occurrence of *Legionella* spp., *L. pneumophila*, and *M. intracellulare*; and the FLA hosts, *Acanthamoeba* spp. and *Vermamoeba vermiformis*, and heterotrophic plate count levels, were monitored, along with chemical water quality, engineering, and operational parameters. Determining how various physical, chemical, and microbial BWS characteristics influence *Legionella* occurrence may elucidate ways to minimize and eliminate their growth within these systems.

## 2. Results

### 2.1. Description of Sampling Locations and Water Quality Characteristics

First draw and second draw (post-flushing) cold bulk water samples and biofilm swab samples were collected every three months (fall, F; winter, W; spring, Sp; and summer, Su) at six locations within a large commercial building (Table 1 and Section 4.1). This building water system (BWS) contained a variety of plumbing materials, varying water flow/rates, and usage patterns at each of the sampled locations (Table 1) with previous detection of *Legionella* in the cold water (data not shown). This building was also chosen because it contains both office and production facilities where large volumes of water are used in the latter portions (e.g., for production processes and cooling) and smaller volumes in the office spaces for employees. Three locations had polyvinyl chloride (PVC) schedule 80 valves that supplied monochloramine-treated water (PVC-MA) or chlorine-treated water with and without passage through the building’s chiller/refrigeration system (PVC-R and PVC-FC, respectively); and the other three sampling locations supplied chlorine-treated water from a cast brass spigot (Spigot), a chrome-plated, forged brass faucet (Faucet), and a drinking water fountain with a stainless steel and ethylene propylene diene bubbler head (Fountain) (Figure S1, Table 1).

The water usage ranged from 5 L to 1.4 million L per season with an average of 278,030 L ± a standard deviation (SD) of 598,785 L per season for each sampling location. The PVC-R location is a high water usage site due to the presence of multiple online and remote water quality sensors that require constant water flow; thus, when this site is excluded, the water usage of the other five locations had an average of 35,194 ± 76,876 L per season. The pipe material from the outlet to the supply feed were either all copper (Spigot, Faucet, and Fountain), all PVC (PVC-MA), a mixture of both (PVC-FC), or a mixture of fiberglass ductile iron and PVC reinforced plastic tubing (PVC-R). 

In this study, a total of 114 bulk water and 57 biofilm samples were collected across the six locations. Bulk water samples were analyzed for pH, turbidity, temperature, and free and total chlorine (Table 2). There were no statistical differences between the pH of the first and second draw samples for each location and sampling time points, but there were differences in pH between the PVC-MA samples and the other five locations (*P* < 0.001). There were no statistical differences between the turbidity of all water samples at each location most likely due to the large range in Nephelometric Turbidity Units (NTUs) observed for each sample (Table 2, Figure S2).

Temperatures between first and second draw samples within each location and time point were also not significant, except for Spigot-first draw versus Spigot-second draw samples (*P* < 0.05). Free and total chlorine levels for first and second draw samples between, and within, each location were not significant, except for Faucet-first draw versus Faucet-second draw and PVC-R-first vs Faucet-first draw samples (*P* < 0.01, Table 2, Figure S3). There were no statistical differences between monochloramine and total chlorine levels in the bulk water from location PVC-MA (*P* > 0.05). There were statistical differences between the heterotrophic plate count (HPC) levels of the first vs second draw samples at only the PVC-R, Spigot, and Faucet locations (*P* < 0.01, Table 2). For biofilm samples, the average HPC level observed at the Faucet location was the highest compared to other locations but was statistically different only from the PVC-R biofilm samples (*P* < 0.01, Table 2).

### 2.2. Legionella Culture Results

Bulk water and biofilm samples were processed and enumerated for *Legionella* colony forming units (CFU) as described in Section 4.2 and Section 4.4. No culturable *Legionella* was detected in samples from the PVC-MA, Spigot, or Fountain location; from the second draw and biofilm samples from PVC-R; and biofilm samples from PVC-FC. However, culturable *Legionella* was detected at various time points for the first and second draw samples from PVC-FC; all sample types from the Faucet; and only the W2018 first draw sample from PVC-R, which contained *Legionella* non-*pneumophila* bacteria as confirmed by colony lysate PCR and latex agglutination (Table 3). 

For the PVC-FC bulk water samples, *Legionella* non-*pneumophila* colonies were detected in both the first and second draw samples during W2018 (4.5 and 2.6 log_10_ CFU 100 mL^−1^, respectively) and W2019 (2.1 and 2.6 log_10_ CFU 100 mL^−1^, respectively) and only the second draw (3.0 log_10_ CFU 100 mL^−1^) during F2018 via colony confirmation PCR (Table 3). The F2018 isolate was identified as *L. feeleii* via indirect immunofluorescent antibody assay as described in Section 4.4. *L. pneumophila* and non-*pneumophila* colonies were detected in the first and second draw samples (3.8 and 2.8 log_10_ CFU 100 mL^−1^, respectively) at this location during Su2018 via colony confirmation PCR. The *L. pneumophila* PCR-positive Su2018 colonies either gave an inconclusive latex agglutination result or were identified as belonging to serogroup (sg) 2–14. Three Su2018 isolates (one from the first draw and two from the second draw samples), one F2018 isolate (from the second draw sample), and four W2019 isolates (two from the first and two from the second draw samples) were processed for whole genome sequencing. One of the two Su2018 second draw isolates was identified as *L. pneumophila* sg5 via indirect immunofluorescent antibody assay.

Throughout the study period, only one second draw (W2019) and one biofilm (F2016) sample, at the Faucet location had culturable *Legionella* bacteria at concentrations of 2.1 log_10_ CFU 100 mL^−1^ and 1.9 log_10_ CFU cm^−2^, respectively (Table 3). The Faucet second draw sample was confirmed as *L. pneumophila* sg1, and the biofilm sample contained a mixture of *L. pneumophila* sg1 and 2–14 colonies as confirmed by PCR and latex agglutination. Fifty percent of the Faucet first draw samples were *Legionella* culture positive: Sp2017, F2017, W2018, Su2018, and W2019 with an average ± SD concentration of 2.0 ± 0.8 log_10_ CFU 100 mL^−1^ (Table 3). *Legionella* identified within these Faucet first draw samples were diverse with only *L. pneumophila* sg1 being identified in the Su2018 and W2019 samples; only *L. pneumophila* sg2–14 in the Sp2017 sample; a mixture of *L. pneumophila* sg1 and 2–14 in the W2018 sample; and *Legionella* non-*pneumophila* identified in the F2017 sample. Five isolates obtained from the Su2018 and one from the W2019 Faucet first draw samples were processed for whole genome sequencing.

For only the Su2018, F2018, and W2019 time points, *L. pneumophila* most probable number (MPN) in bulk water and biofilm samples were enumerated using Legiolert^®^ as described in Section 4.4. All samples at each location had no detectable *L. pneumophila* MPN except for the Faucet first draw (Su2018 1.8 log_10_ MPN 100 mL^−1^; W2019 3.8 log_10_ MPN 100 mL^−1^) and second draw (Su2018 1.4 log_10_ MPN 100 mL^−1^; W2019 2.5 log_10_ MPN 100 mL^−1^) samples. Four wells from the first draw and three wells from the second draw Legiolert^®^ tray, read as *L. pneumophila* positive, were sampled and pure colonies were obtained as described in Section 4.4. Isolates were *Legionella* and *L. pneumophila* PCR-positive but gave an inconclusive latex agglutination result. One of the W2019 Faucet second draw Legiolert^®^ isolates was processed for whole genome sequencing and identified as sg5 via indirect immunofluorescent antibody assay.

### 2.3. Detection of Water-based Pathogens and Free-living Amoebae (FLA) via Quantitative PCR Analyses

#### 2.3.1. Legionella spp. and L. pneumophila Occurrence

**Figure 1:**
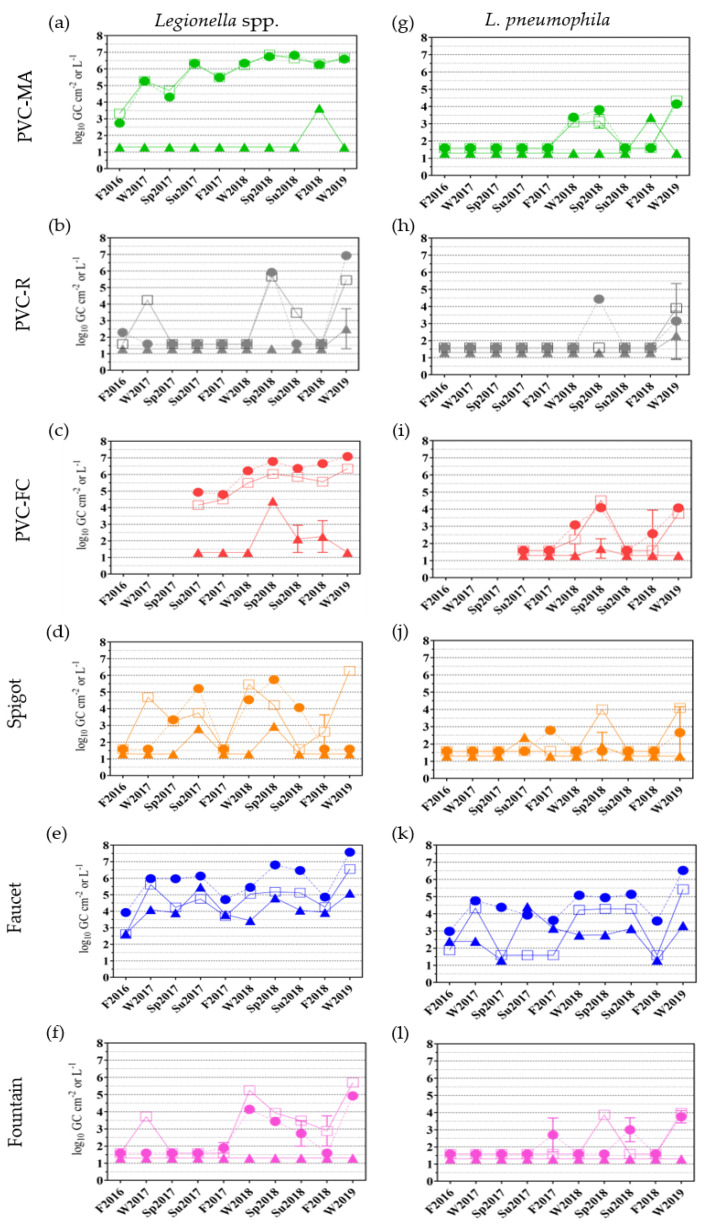
qPCR detection of *Legionella* at six locations within a building water system. Bulk water (first draw, filled circles ●; second draw, open squares □) and biofilm (filled triangles ▲) samples were analyzed by *Legionella* spp. (**a**–**f**) and *L. pneumophila* (**g**–**l**) 16S rRNA qPCR as described in Section 4.6 from each of the six locations listed on the left. Each data point is the mean of duplicate wells with standard deviation. No sampling occurred in F2016–Sp2017 for location PVC-FC. The limit of detection for bulk water and biofilm samples were 1.6 log_10_ genomic copies (GC) L^−1^, and 1.3 log_10_ GC cm^−2^, respectively.

In addition to culture methods, bulk water and biofilm samples were also analyzed for the presence of *Legionella* spp. and *L. pneumophila* via qPCR as described in Section 4.6. In agreement with the specificity and sensitivity of the *Legionella* genus and *L. pneumophila* species-specific qPCR assays used in this study, the total *Legionella* levels detected in the bulk water and biofilm samples were higher than the *L. pneumophila* levels observed in the corresponding samples (Figure 1a–f and g–l). The presence of *Legionella* and *L. pneumophila* was detected at all sampling locations and sample types and within each location, detection generally occurred more frequently, and at higher levels, in the bulk water compared to the biofilm samples (Figure 1, first draw, circles, and second draw, squares, compared to biofilm, triangles). 

*Legionella* was detected in all bulk water samples at locations PVC-MA, PVC-FC, and Faucet (Figure 1a, c, and e). In contrast, only 30% (3/10 first draw; 3/10 second draw) of PVC-MA, 50% (4/7 first draw; 3/7 second draw) of PVC-FC, and 80% (10/10 first draw; 6/10 second draw) of Faucet samples were *L. pneumophila* positive (Figure 1g, i, and k). *Legionella* was detected sporadically in the bulk water samples at locations PVC-R (35%, 3/10 first draw; 4/10 second draw), Spigot (60%, 5/10 first draw; 7/10 second draw), and Fountain (55%, 5/10 first draw; 6/10 second draw) (Figure 1b, d, and f). *L. pneumophila* was detected in 15% (2/10 first draw; 1/10 second draw) of the PVC-R, in 20% (2/10 first draw; 2/10 second draw) of the Spigot, and in 25% (3/10 first draw; 2/10 second draw) of the Fountain bulk water samples (Figure 1h, j, l).

Biofilm samples from all locations had sporadic detection of *Legionella* and *L. pneumophila* except for the Faucet and Fountain locations (Figure 1, triangles). At the Faucet location, both were detected in all biofilm samples except for the Sp2017 and F2018 time points for *L. pneumophila* (Figure 1e and f, blue triangles). At the Fountain location, there was no detectable *Legionella* or *L. pneumophila* at all time points (Figure 1f and l, pink triangles). Additionally, for the biofilm samples, *Legionella* was detected in only one PVC-MA (F2018) and PVC-R (W2019); three PVC-FC (Sp2018, Su2018, and F2018); and two Spigot (Su2017 and Sp2018) biofilm samples (Figure 1a–d, triangles). Similarly, *L. pneumophila* was detected in one PVC-MA (F2018), PVC-R (W2019) and only one of the three *Legionella* positive PVC-FC (Sp2018) samples; and two Spigot (Su2017 and Sp2018) biofilm samples (Figure 1g–j, triangles).

#### 2.3.2. Free-living Amoebae and *Mycobacterium intracellulare* Occurrence

**Figure 2:**
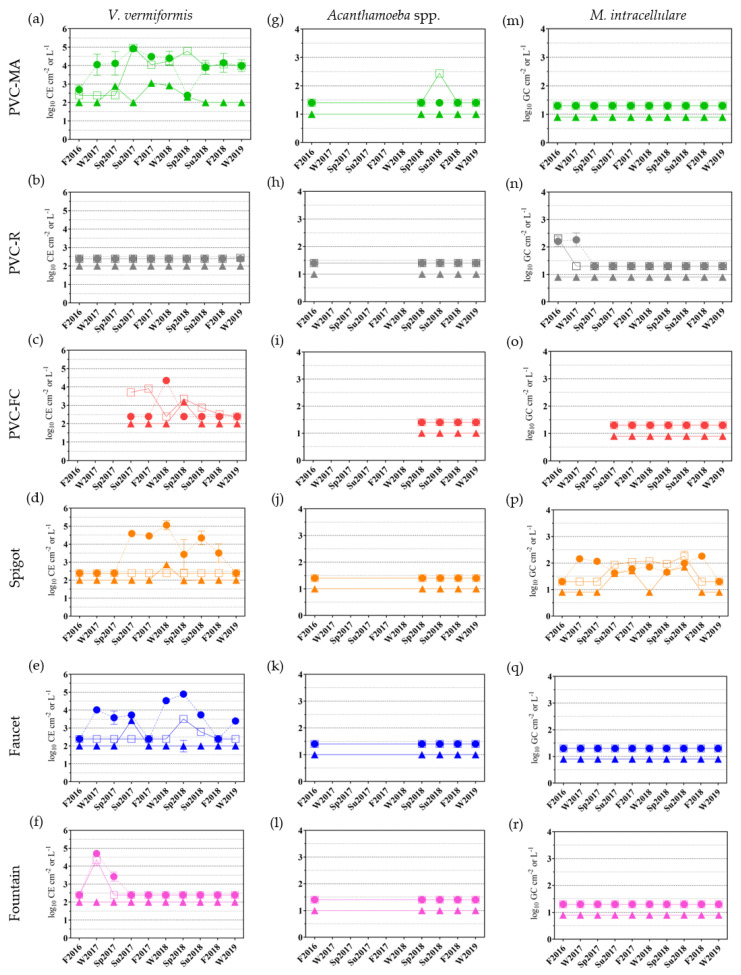
qPCR detection of free-living amoeba and *Mycobacterium intracellulare*. Bulk water (first draw, filled circles ●; second draw, open squares □) and biofilm (filled triangles ▲) samples were analyzed for *Vermamoeba vermiformis* (**a**–**f**), *Acanthamoeba* spp. (**g**–**l**), and *M. intracellulare* (**m**–**r**) by qPCR as described in Section 4.6 from each of the six locations listed on the left. Each data point is the mean of duplicate wells with standard deviation. No sampling occurred from fall (F) 2016 to spring (Sp) 2017 for location PVC-FC. The limit of detection for bulk water and biofilm samples were 2.4 log_10_ cell equivalents (CE) L^−1^ and 2.0 log_10_ CE cm^−2^ for the *V. vermiformis*; 1.4 log_10_ CE L^−1^ and 1.0 log_10_ CE cm^−2^ for the *Acanthamoeba* spp.; and 1.3 log_10_ GC L^−1^ and 0.9 log_10_ GC cm^−2^ for *M. intracellulare* assays, respectively.

To determine if *Legionella* occurrence correlated with the presence of their eukaryotic hosts and another water-based human opportunistic pathogen, bulk water and biofilm samples at each location were analyzed for two different free-living amoebae, *Vermamoeba vermiformis* and *Acanthamoeba* spp., and *Mycobacterium intracellulare* (Figure 2). *V. vermiformis* was detected in the bulk water and biofilm samples at various time points for locations PVC-MA, PVC-FC, Spigot, and Faucet; in only the bulk water and not biofilms for the Fountain location; and was not detected in any samples from location PVC-R (Figure 2a–f). For *Acanthamoeba* spp., only five time points were analyzed for each location (F2016, Sp2018 to W2019), except for PVC-FC where only four were analyzed (Sp2018 to W2019) (Figure 2g–l). Notably, only the second draw sample at location PVC-MA had detectable *Acanthamoeba* spp. (Figure 2g, green square mean ± SD of 2.4 ± 0.0 log_10_ CE L^−1^).

At location PVC-MA, *V. vermiformis* was detected in 80% of the bulk water samples (9/10 first draw; 7/10 second draw) and 40% of the biofilm samples (4/10). *V. vermiformis* was detected in 43% of the bulk water samples at the PVC-FC (1/7 first draw; 5/7 second draw); 30% at the Spigot (6/10 first draw; 0/10 second draw); and 45% at the Faucet (7/10 first draw; 2/10 second draw) locations (Figure 2c–e). Only one biofilm sample was positive for *V. vermiformis* at the PVC-FC (Sp2018), Spigot (W2018), and Faucet (Su2017) locations (Figure 2c–e). For the Fountain location, *V. vermiformis* was detected in 15% (2/10 first draw; 1/10 second draw) of the bulk water samples (Figure 2f). All samples were negative for *M. intracellulare* at locations PVC-MA, PVC-FC, Faucet, and Fountain (Figure 2m, o, e–f). At location PVC-R, only 15% (2/10 first draw; 1/10 second draw) of water samples were positive (Figure 2n). *M. intracellulare* was more frequently detected in the Spigot bulk water (65%, 8/10 first draw; 5/10 second draw) and biofilm (40%, 4/10) samples.

### 2.4. Additional Sampling Sites

During the W2019 time point, 1 L of first draw samples were collected at two additional locations within the large BWS: an outlet off of the incoming main water line located in the boiler room (BWS Supply Line) and a recirculating pipe loop (PVC-Loop) previously described [32]. The bulk water sample from the BWS Supply Line had a turbidity of 0.34 NTU; 0.00/0.05 mg L^−1^ free/total chlorine; pH of 8.53; temperature of 39.5 °C; and HPC levels of 1.6 log_10_ CFU 100 mL^−1^. For the PVC-Loop, the bulk water sample had a turbidity of 0.21 NTU; 0.01/0.01 mg L^−1^ free/total chlorine; pH of 8.31; temperature of 20.6 °C; and HPC levels of 4.7 log_10_ CFU 100 mL^−1^.

The BWS Supply Line contained 3.0 log_10_ CFU and MPN 100 mL^−1^ of *L. pneumophila* sg1 and 2–14 as confirmed by colony lysate PCR and latex agglutination. Two colonies were *Legionella* and *L. pneumophila* PCR positive, identified as sg1, and processed for whole genome sequencing. The BWS Supply Line sample was negative for *Acanthamoeba* spp. and *M. intracellulare* but contained 3.9 log_10_ CE 100 mL^−1^ of *V. vermiformis*, 7.3 log_10_ GC 100 mL^−1^ of *Legionella* spp., and 6.9 log_10_ GC 100 mL^−1^ of *L. pneumophila*. The PVC-Loop bulk water sample was negative for *V. vermiformis* and *M. intracellulare* but contained 3.0 log_10_ CE 100 mL^−1^ of *Acanthamoeba* spp., 7.4 log_10_ GC 100 mL^−1^ of *Legionella* spp., and 7.3 log_10_ GC 100 mL^−1^ of *L. pneumophila*. 

The PVC-Loop had a high level of non-*Legionella* background that negatively impacted the enumeration of presumptive *Legionella* colonies. Although *Legionella* CFU could not be determined, 3.4 log_10_ MPN 100 mL^−1^ of *L. pneumophila* was detected using Legiolert^®^. Four wells of the PVC-Loop Legiolert^®^ tray, read as *L. pneumophila* positive following manufacturer’s protocols, were sampled, and pure colonies were obtained as described in Section 4.4. Legiolert^®^ isolates were analyzed by PCR and latex agglutination. One of the four Legiolert^®^ isolates was *Legionella* and *L. pneumophila* PCR negative and processed for whole genome sequencing. Three of the four Legiolert^®^ isolates were *Legionella* and *L. pneumophila* PCR positive with 2/3 giving an inconclusive agglutination result and 1/3 identified as sg5. This PCR positive, *L. pneumophila* sg5 isolate was processed for whole genome sequencing. 

### 2.5. Statistical Correlations between Water Quality Characteristics

**Figure 3:**
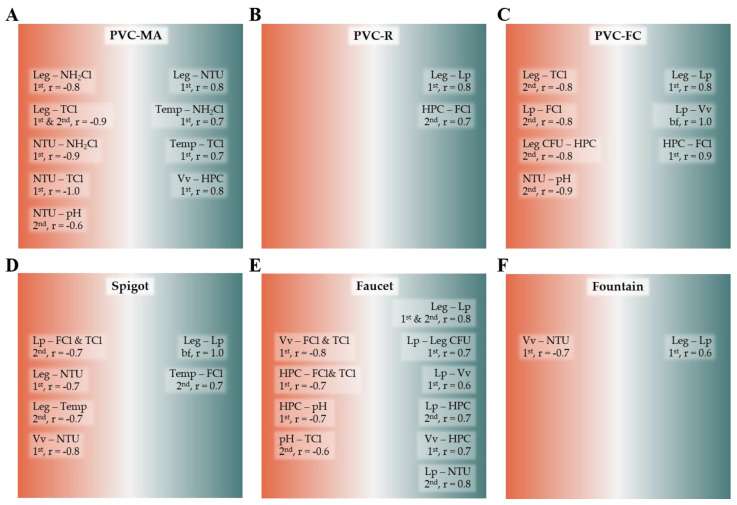
Negative and positive correlations between water quality characteristics. Pairwise comparisons with statistically significant and strong correlations are shown for each location (**A**: PVC-MA; **B**: PVC-R; **C**: PVC-FC; **D**: Spigot; **E**: Faucet; **F**: Fountain) and sample type. Negative and positive correlations are shown on the left and right half of each square, respectively. Abbreviations: 1^st^, first draw; 2^nd^, second draw; bf, biofilm; FCl, free chlorine; HPC, heterotrophic plate count; Leg, *Legionella* spp.; Leg CFU, culturable *Legionella*; Lp, *L. pneumophila*; NH_2_Cl, monochloramine; NTU, turbidity; pH, potential of hydrogen; TCl, total chlorine; Vv, *V. vermiformis*.

Correlation analysis was performed, as described in Section 4.8, to determine if there were negative or positive associations between the observed water quality characteristics within each sampling location. As expected, there was a strong positive correlation between free and total chlorine in both the first and second draw bulk water samples at all locations supplied with chlorinated water, (r = 0.7–1.0, *P* < 0.05). For the other pairwise comparisons within each location, only the statistically significant (*P* < 0.05) correlations between water quality characteristics are shown in Figure 3.

*Legionella* and/or *L. pneumophila* occurrence was negatively correlated with disinfectant residual at three of the six sampling locations. *Legionella* spp. was negatively correlated with NH_2_Cl (r = −0.8) and TCl (r = −0.9) in the first draw, but only with TCl in the second draw samples (r = −0.9), at the PVC-MA location. At the PVC-FC location, *Legionella* spp. and *L. pneumophila* were negatively correlated with TCl and free chlorine (FCl), respectively, only in the second draw samples (r = −0.8, *P* < 0.05). Similarly, *L. pneumophila* was negatively correlated to both FCl and TCl in the Spigot second draw samples (r = −0.7). Notably, for the Faucet location that had consistent levels of *Legionella* spp. and *L. pneumophila* (Figure 1e and k), as well as culturable *Legionella* (Table 3), no statistical correlations were made between *Legionella* and disinfectant residual (Figure 3, Faucet). Moreover, occurrence of *V. vermiformis* was also negatively associated with FCl and TC (r = −0.8) at only the Faucet location.

At all sampling locations except for PVC-MA, *Legionella* spp. was positively correlated with *L. pneumophila* in the first draw, second draw, and biofilm samples depending on the location (Fountain, r = 0.6; PVC-R, PVC-FC, and Faucet, r = 0.8; and Spigot, r = 1.0). Additionally, at the Faucet location, culturable *Legionella* was positively correlated with *L. pneumophila* detection in the first draw water samples (r = 0.7). For *L. pneumophila* and *V. vermiformis*, a positive correlation was found at locations PVC-FC (biofilm, r = 1.0) and Faucet (first draw, r = 0.6). Both positive and negative correlations between HPCs and chlorine residuals were observed at three of the six locations. There were positive correlations between HPC and FCl in the first draw (PVC-FC, r = 0.9) and second draw (PVC-R, r = 0.7) samples and negative correlations between HPC and both FCl and TCl in the first draw samples at the Faucet location (r = −0.7). At the Faucet location, HPCs were negatively correlated with pH (r = −0.7) in the first draw samples. HPCs were also negatively associated with culturable *Legionella* in the PVC-FC second draw samples (r = −0.8), but positively associated with *L. pneumophila* molecular detection in the Faucet second draw samples (r = 0.7). Only a positive correlation was found between HPCs and *V. vermiformis* detection in the first draw samples at two locations, PVC-MA (r = 0.8) and Faucet (r = 0.7).

There were strong correlations between *Legionella* detection and various physiochemical water quality parameters. *Legionella* spp. was negatively correlated to temperature at only one location (Spigot, second draw, r = −0.7). Turbidity (NTU) was positively correlated with *L. pneumophila* detection in the Faucet second draw samples (r = 0.8); however, both positive and negative correlations were observed between NTU and *Legionella* spp. detection at two separate locations PVC-MA (first draw, r = 0.8) and Spigot (first draw, r = −0.7), respectively. Only a negative correlation was found between NTU and *V. vermiformis* detection in the first draw samples at two locations, Spigot (r = −0.8) and Fountain (r = −0.7).

Correlations between the physiochemical water quality parameters included a positive association between temperature and both NH_2_Cl and FCl at the PVC-MA (first draw, r = 0.7) and between temperature and FCl at the Spigot (second draw, r = 0.7) locations, respectively. There was a negative correlation between NTU and pH in the second draw samples at the PVC-MA (r = −0.6) and PVC-FC (r = −0.9) locations; and negative correlation between NTU and NH_2_Cl (r = −0.9) and TCl (−1.0) in the first draw samples at the PVC-MA location.

### 2.6. Whole Genome Sequencing of Drinking Water Isolates

**Figure 4:**
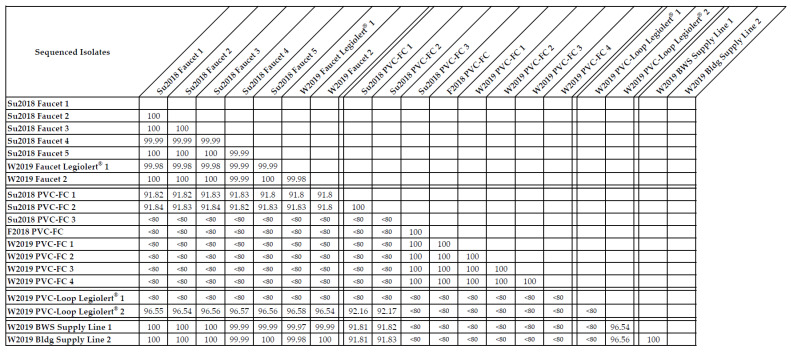
Average nucleotide identity (ANI) matrix for the whole-genome sequenced isolates

Nineteen representative *L. pneumophila* and non-*pneumophila* isolates from the Su2018, F2018, and W2019 time points and PVC-FC, Faucet, BWS Supply Line, and PVC-Loop locations were submitted for whole genome sequencing as described in Section 4.7 (Table 4). All seven Faucet isolates from the Su2018 (Faucet 1–5) and W2019 (Faucet Legiolert^®^ 1 and Faucet 2) time points, and the W2019 BWS Supply Line 1 and 2 isolates, were identified as *L. pneumophila* Sequence Type (ST) 1 with an average genome size of 3.6 million base pairs (Mbp), a guanine–cytosine (G + C) content of 38%, and approximately 3200 predicted genes. The average nucleotide identity (ANI) between these seven Faucet and two BWS Supply Line isolates was between 99.97% and 100% indicating that these isolates were the same *L. pneumophila* strain (Figure 4). These isolates were also identified as sg1 via latex agglutination as described in Section 2.2 and 2.4.

Of the eight PVC-FC isolates, two (Su2018 PVC-FC 1–2) were identified as *L. pneumophila* ST2037, with a comparable genome size (~3.5 Mbp), 38% G + C content, and 100% ANI (Table 4, Figure 4), with Su2018 PVC-FC 2 identified as *L. pneumophila* sg5 via indirect immunofluorescent antibody assay as described in Section 2.2. The remaining six PVC-FC isolates (Su2018 PVC-FC 3, F2018 PVC-FC, and W2019 PVC-FC 1–4) were identified as *Legionella* with a genome size of 3.0–3.3 Mbp, 41% G + C content, and 99.99–100% ANI (Table 4, Figure 4), with F2018 PVC-FC identified as *L. feeleii* via indirect immunofluorescent antibody assay. The W2019 PVC-Loop Legiolert^®^ 1 isolate, derived from a *L. pneumophila* positive well, was identified as *Ochrobactrum* with a higher genome size of 4.7 Mbp, higher G + C content of 67% compared to the W2019 PVC-Loop Legiolert^®^ 2 isolate identified as *L. pneumophila* ST1950, with a genome size of 3.4 Mbp, 38% G + C content, and 3057 predicted genes (Table 4). 

**Figure 5:**
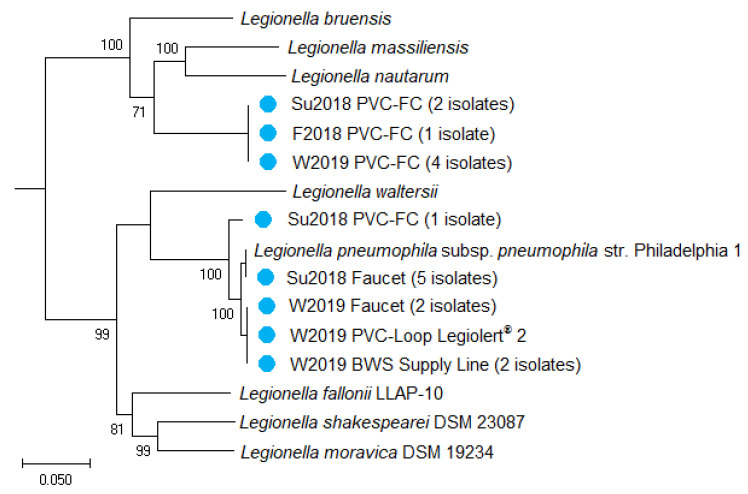
Phylogenetic tree illustrating isolate relatedness to reference genomes. Representative strains (**blue dots**) from each sampling location and time were chosen for construction of this phylogenetic tree. Numbers presented are confidence values (**bootstrapping**) used by FastTree 2 to estimate maximum likelihood. The scale bar represents 0.05 nucleotide substitutions per site.

Between the *L. pneumophila* sg5 ST1950 isolate and sg5 ST2037 isolates, the ANI was 92%, suggesting that they belong to the same species, but are different strains (Figure 4). Notably, the nine *L. pneumophila* sg1 ST1 isolates had a higher ANI of 96% with the *L. pneumophila* sg5 ST1950 isolate compared to the 92% ANI with the *L. pneumophila* sg5 ST2037 isolates. This higher nucleotide similarity between the *L. pneumophila* sg1 ST1 and *L. pneumophila* sg5 ST1950 can be visualized with their clustering in the phylogenetic tree (Figure 5). The *L. pneumophila* sg1 ST1 strains (W2019 BWS Supply Line, Su2018 and W2019 Faucet) were in the same branch as the *L. pneumophila* sg5 ST1950 strain, while the *L. pneumophila* sg5 ST2037 strain (Su2018 PVC-FC) was in a separate branch. The PVC-FC isolates were identified as *L. feeleii* via indirect immunofluorescent antibody assay and sequence similarities to two *L. feeleii* reference genomes (strain WO-44C and NCTC11978) which displayed >99.9% similarity based on 16S rRNA and ANI of 98.5%. Figure 5 shows the *L. feeleii* isolates in their own cluster with the reference genomes of *L. massiliensis* and *L. nautarum* in the next closest branch. The two *L. feeleii* reference genomes were not included in the sequence database at the time of this analysis and thus are not represented in the phylogenetic tree (Figure 5).

## 3. Discussion

The main objective of this study was to gain a better understanding of water quality parameters that correlated with *Legionella* occurrence within a large complex building water system (BWS) over an extended period of time. Cold bulk water and biofilm samples were collected to monitor the occurrence and distribution of *Legionella*, other water-based pathogens, and eukaryotic hosts, while considering seasonal fluctuations, and varying engineering, operational, and water quality characteristics. Cold water was analyzed in this study given (1) the high prevalence of *Legionella* contamination previously identified in cold water samples within public building, private residences, healthcare facilities, and water storage tanks; (2) *Legionella* transmission and infections epidemiologically linked to cold water exposure; and (3) the current recommendations to monitor both hot and cold water in BWS to control for *Legionella* [33]. In this study, both culture and molecular methods were used for *Legionella* detection and culture isolates were submitted for whole genome sequencing for further genetic characterization. 

Two percent of biofilm samples (1/57) and 12% of bulk water samples (14/114) were culture-positive for *Legionella* of which 57% (8/14) were isolated during the winter; 21% (3/14) during the summer; 14% (2/14) during the fall; and 7% (1/14) during the spring. Legionnaires’ disease (LD) cases typically peak during the summer and fall seasons [34,35]. However, other epidemiological studies reported a winter peak for non-travel related cases [36] and no monthly or seasonal correlations for nosocomial-acquired [37] and community-acquired *Legionella* pneumonia cases [38]. Higher incidences of legionellosis have been associated with various meteorological factors (e.g., humidity, temperature, rainfall, atmospheric pressure); geographic location; and properties of the local watershed and source water (e.g., proximity and water temperature, levels, and flow) [39,40,41,42]. Thus, the exact environmental mechanisms and triggers of *Legionella* transmission and subsequent disease incidences are yet to be clearly defined.

Culture and qPCR are frequently used methods for *Legionella* environmental detection [43,44,45]. However, due to discrepancies between and within each of these methods, interpretations of *Legionella* occurrence across various studies and correlations to legionellosis health risks within BWSs are challenging [46]. Moreover, false positivity rates for the Legiolert^®^ method have been reported to be between 0% and 3.3% for potable water and 4.9% and 11% for non-potable water [47,48,49]. Such as with *Ochrobactrum* identified in the PVC-Loop location, stable colonization of a Legiolert^®^ false positive causing strain at an environmental monitoring site may continually overestimate *L. pneumophila* levels and confound interpretations of their occurrence at those sites. Culture-based methods are a reliable indicator of pathogen viability and potential health risks associated with *Legionella* detection; however, periodic validation of culture-based results can be performed using molecular methods. 

In this study, the PVC-R W2018 first draw sample was *Legionella* culture positive, but *Legionella* qPCR negative (Table 3, Figure 1b), while the PVC-FC Su2018 second draw sample was *Legionella* and *L. pneumophila* culture positive, but only *L. pneumophila* qPCR negative (Table 3, Figure 1c,i). Culture-positivity/qPCR-negativity has been observed previously and was associated more with drinking water compared to cooling tower water samples [50,51,52]. Furthermore, *L. pneumophila* qPCR levels were higher than those of *Legionella* spp. in four first draw samples (Spigot F2017, W2019 and Fountain F2017, Su2018) (Figure 1d, j and f, l) and *L. pneumophila* was also observed in hot water, but not cooling tower water samples [53]. These discrepancies have been attributed to the presence of PCR inhibitors and competing non-target organisms, and varying culture conditions (e.g., growth temperature, agar type) and sample processing steps that may select for, or inhibit growth of, different strains of *Legionella* [46].

For *Legionella* environmental monitoring, samples that can be collected for analysis include (1) biofilm materials, which contain a concentration of surface attached microorganisms; (2) first draw, stagnant water samples, which represent water quality at the outlet; and (3) second draw, post-flushed samples, which represent water quality supplied to the outlet from within the building water system [54,55]. Of the *Legionella pneumophila* and non-*pneumophila* culture-positive bulk water samples, 64% (9/14) were first draw and 36% (5/14) were second draw samples, while only 2% (1/57) of biofilm samples were culture-positive (Table 3). From the qPCR analyses, *Legionella* was detected in 30% (17/57) of biofilm samples and 74% (84/114) of bulk water samples of which 48% (40/84) were first draw and 52% (44/84) were second draw samples. *L. pneumophila* was detected in 23% (13/57) of biofilm samples and 36% (41/114) of bulk water samples of which 59% (24/41) were first draw and 41% (17/41) were second draw samples (Figure 1). 

Differences in physiochemical and microbial water quality parameters between first and second draw samples were previously reported with microbial loads generally higher in the first draw depending on the sampling location and volume collected [56,57]. As stated in Section 2.1, there were no statistical differences in pH, temperature, free chlorine, monochloramine, and total chlorine between the first and second draw samples at all locations with the exceptions of temperature for the Spigot samples and free and total chlorine for the Faucet samples. Three of the six locations had statistical differences between the first and second draw samples for HPCs: PVC-R, Spigot, and Faucet. For *Legionella* levels detected by qPCR, there were strong positive correlations between the first and second draw samples from all locations: PVC-MA (r = 0.9, *P* < 0.001), PVC-R (r = 0.6, *P* < 0.05), PVC-FC (r = 0.9, *P* < 0.01), Faucet (r = 0.8, *P* < 0.01), and Fountain (r = 0.8, *P* < 0.01), except for the Spigot location (r = 0.2, *P* = 0.575). For *L. pneumophila* levels as detected by qPCR, there were only strong positive correlations between the first and second draw samples at three of the six locations: PVC-MA (r = 1.0, *P* < 0.001), PVC-FC (r = 0.9, *P* < 0.01), and Faucet (r = 0.7, *P* < 0.05). 

The results between the bulk water samples suggested that an analysis of either the first or second draw samples was able to indicate the presence of *Legionella*. Moreover, it is unclear why only the Faucet location showed statistical differences of both free and total chlorine between the first and second draw samples as water usage at this site was higher than two other locations and the distance of the Faucet outlet was greater than and almost equal to three other sampling locations (Table 1). The only difference between the Faucet location and all others was the ability to draw hot water from this outlet, but only cold water was analyzed in this study; thus, hot water may be contributing to bacterial contamination in the Faucet.

After conversion to monochloramine disinfection within BWSs, there were reductions in the number of distal sites testing positive for *Legionella* (39–100% to 0–18% positivity); however, there was a large range in the log reduction of *Legionella* levels (0.2 to 3 log_10_ CFU L^−1^) with one study reporting no changes in levels post-conversion during the one- to three-year monitoring period [58,59,60]. Control of biofilm-associated *Legionella* was also observed [58,61], most likely due to the better penetration of monochloramine into biofilms compared to chlorine [62]; however, control of biofilm-associated *L. pneumophila* (Lp) was previously reported to be pipe material specific during chlorine and monochloramine treatment [63]. The PVC-MA location used in this study has been operating with a monochloramine residual for approximately 10 years with a two-month chlorine conversion from December 2013 to February 2014. During the sampling period, no culturable *Legionella* was detected, but molecular analyses indicated a consistent and high level of *Legionella* and sporadic detection of *L. pneumophila* in the bulk water and biofilm samples. Specifically, 100% (20/20) of the bulk water samples and 10% (1/10) of biofilm samples had detectable *Legionella*; while *L. pneumophila* was detected in 30% (6/20) of bulk water samples (three first draw and three second draw samples) and 10% (1/10) of biofilm samples (Figure 1a and g). Using *E. coli*, monochloramine was shown to disrupt protein-mediated metabolic processes with no damage to the cell envelope or nucleic acids [64]. This finding is supported by other studies demonstrating no significant impacts on *Legionella* 16S rRNA gene transcript levels during monochloramine versus chlorine treatment [63] and *Pseudomonas aeruginosa* extracellular polymeric substances material limiting and delaying monochloramine access to the cell surface [65]. Thus, it is unclear what the exact mechanisms are for monochloramine control of *Legionella* bacteria and whether different surface properties (e.g., lipopolysaccharides used for *L. pneumophila* serogroup identification) would result in varying degrees of inactivation with intermediate stages allowing for *Legionella* regrowth.

Numerous studies have reported more frequent detection of *V. vermiformis* within BWSs compared to *Acanthamoeba* spp. [56,66,67]. Concordantly, *Acanthamoeba* spp. were undetectable in the bulk water and biofilm samples during F2016, thus the following five sampling time points were excluded; however, the analysis was resumed in Sp2018 to confirm the low frequency and/or undetectable observation for *Acanthamoeba* spp. in BWS samples (Figure 2g–l). *Acanthamoeba* spp. was detected in only one second draw sample at the PVC-MA location (Figure 2g). Similarly, *M. intracellulare* was infrequently detected in this study (Figure 2n and p) as previously observed for water samples from chlorinated BWSs [23,68]. *V. vermiformis* was detected at all locations except PVC-R (Figure 2a–f) with positive correlations between *L. pneumophila* and *V. vermiformis* in PVC-FC biofilms and Faucet first draw samples. Given that FLA detection has been correlated to *Legionella* and *Mycobacterium* in drinking water systems [69], there is utility in monitoring for FLA to better understand the conditions and FLA members contributing to *Legionella* and mycobacterial survival within BWSs. 

Although diverse populations of *Legionella* bacteria have been described in drinking water [70,71], environmental monitoring of BWSs focuses primarily on *L. pneumophila* detection since this species makes up the majority of clinical isolates, with > 80% of those isolates belonging to serogroup (sg) 1 [72,73]. Serotyping allows for the differentiation of *L. pneumophila* isolates based on their reactivity to the Dresden Panel of antibodies that recognize distinct structures on the bacterial lipopolysaccharide molecule [74,75]. *L. pneumophila* contains 17 serogroups and 10 subgroups within sg1 [76,77]. The latex agglutination serotyping method used in this study has been shown to produce false negatives as *Legionella*-like colonies isolated from drinking water samples, confirmed as *L. pneumophila* via 16S rRNA sequence analyses, were agglutination negative (this study; [78]). Thus, molecular-based methods, such as sequence-based typing (SBT) and whole genome sequencing (WGS), are increasingly being used due to their reliability, better resolution, and discriminatory power for describing genetic diversity, environmental distribution, evolution, population structure, clonal expansion, and virulence properties of *Legionella* isolates [77,79]. 

In this study, *L. feeleii*, *L. pneumophila* sg1 sequence-type (ST) 1, and *L. pneumophila* sg5 ST 1950 and ST2037 were identified via 16S rRNA-based analyses, serotyping (latex agglutination and indirect immunofluorescent antibody assays), WGS, and SBT analyses. These unique *Legionella* strains were isolated from the Faucet, PVC-FC, PVC-Loop, and BWS Supply Line locations during the Su2018, F2018, and W2019 time points (Table 4 andFigure 4). ST1 is the most commonly identified and globally distributed strain isolated from both environmental and clinical samples ([79,80]; this study). Identification of previously unknown STs (e.g., ST1950 and ST2037 from this study) supports the continued SBT of clinical and environmental isolates. This will help elucidate which STs may be more globally distributed or geographically confined, and which are more associated with disease cases, such that when those are environmentally identified, preventative measures can be implemented to limit public health and exposure risks to these pathogens. WGS analyses provide more detailed genetic information about the *Legionella* strain, beyond those obtained from SBT alone, enabling potential subspecies identification, refined taxonomic classification, and genetic profiling for virulence properties [77]. *Legionella* diversity and distribution data may also reveal environmental parameters that influence *Legionella* occurrence and survival within specific environments such as BWSs.

Due to lengthy incubation periods required for *Legionella* culture, the tendency for qPCR to overestimate their levels, and the discrepancies associated with these methods, as described above, use of other microbial and/or physiochemical water quality parameters as potential indicators for *Legionella* presence in BWSs has been investigated [70]. As described in Section 2.5, there were strong statistical correlations observed between various water quality parameters and *Legionella* occurrence such as *V. vermiformis*, HPC, chlorine residual, temperature, and turbidity (Figure 3). However, these correlations were location and sample type specific with conflicting positive and negative correlations for turbidity and HPC. Conflicting correlations between the latter and *Legionella* levels have been reported previously with either strong correlations [81], no correlations [82,83,84,85], or possible seasonal dependencies for these correlations [7]. Other conflicting correlations were reported between *Legionella* occurrence and pH, temperature, various minerals and metals (Ca, Cu, Fe, Mg, Mn, and Zn), total organic carbon, conductivity, and free chlorine at the sampling site and building supply feed [85,86,87]. 

Thus, further analyses of existing data and correlations to *Legionella* occurrence, as well as more in-depth studies on identifying these correlations, need to be performed. This will enable determinations as to whether correlations are dependent on complex factors, either individually or in concert, such as disinfectant type; source water quality fluctuations; genetic background of detected *Legionella* populations; presence of other drinking water microorganisms; or certain engineering and operational water system aspects specific to sampled locations. The notable observations from this study were (1) detection and culture of *Legionella* from outlets that neither supply, nor are connected to plumbing for, hot water; (2) isolation of diverse *L. pneumophila* and non-*pneumophila* strains from different locations; (3) utility of whole genome sequencing and sequence-based typing for enhanced isolate description and characterization of their distribution; (4) *Legionella* levels detected during monitoring can significantly differ between the first and second draw sample; and (5) negative and positive correlations between *Legionella* and various water quality parameters were location and sample type specific. 

Confoundingly, occurrence alone is not the most important factor for legionellosis risk. Environmental, bacterial, and host specific factors such as aerosolization into respirable droplets and their potential for human exposure; virulence of the environmental *Legionella* strain; and host immune status and susceptibility to infection, collectively play an important role in exposure risks and disease outcome. The main objective of this study was to gain a better understanding of *Legionella* occurrence and water quality parameters supporting their growth within a large, complex building water system. Information from this, and future studies, will help elucidate ways to effectively manage the risks associated with *Legionella* exposure within these drinking water distribution systems.

## 4. Materials and Methods 

### 4.1. Sampling Locations

A 40-year old, 33,000 square ft. building with an average potable water usage of 3.6 million gallons (13.6 million L) per year was used in this study. Water usage at any given location varies widely depending upon the activity (floor washing, water storage tank cleanout, etc.) being conducted and the facility cooling demand during warmer months. The building’s potable water supply is derived from river water treated by coagulation, flocculation, and sedimentation; followed by sand, gravel, and granular activated carbon filtration; and then chlorination. Cold potable water samples were collected seasonally every three months from six locations throughout the building (Table 1, Figure S1). The total number of samples for each site was 30 (10 first and second draw bulk water and 10 biofilm samples) collected over a 28-month period, October 2016 to February 2019; except for site PVC-FC, where the total number of samples was 21 (seven first and second draw bulk water and biofilm samples) collected over an 18-month period, August 2017 to February 2019. Sampling time points are denoted F, for fall; W, for winter; Sp, for spring; and Su, for summer followed by the corresponding year. F, W, Sp, and Su samples were collected during the months of October, November, February, May, and August, respectively. 

Within this building, a semi-closed pipe loop distribution system simulator was fed with the chlorinated municipal drinking water, described above, and amended with ammonium hydroxide and sodium hypochlorite (Sigma Aldrich, St. Louis, MO, USA) to yield a 2 mg L^−1^ monochloramine residual as previously described [88]. Average monochloramine and ammonia levels (± SD) during this sampling period were 1.25 ± 0.37 and 0.16 ± 0.07 ppm, respectively.

### 4.2. Sample Collection and Processing

For each sampling location, the first draw sample was taken immediately after turning the tap on, while the second draw was collected after 10 s of flushing (approximately 4 L), except for Fountain where the second draw sample was collected after 30 s of flushing (approximately 2 L). The 10–30-second flush time was used to ensure collection of non-stagnant water that was still representative of water quality conditions within the BWS. Sampling took place early in the morning after an overnight stagnation period. Water samples were collected in sterile 1 L plastic bottles and 1 mL of 10% w/v sodium thiosulfate was added to neutralize any disinfectant residual. An additional 100 mL was also collected for water quality analysis as described below. Approximately 1 L of each bulk water sample was filtered through a 0.2 μm polyethersulfone membrane (Supor^®^ Membrane, PALL Life Sciences, Nassau, NY, USA). Filters were placed into 11 mL of UV-light dechlorinated, 0.22 μm filtered drinking water (dfH_2_O), and vortexed at maximum speed for 1 min to resuspend the concentrated bulk water material. For biofilm collection, a sterile polyester tipped applicator was used to swab an approximate area of 2 cm^2^ inside the tap. The applicator was then placed in a 14-mL round bottom tube containing 2 mL of dfH_2_O and vortexed vigorously for 1 min to resuspend the collected biofilm material. 

Approximately 1 mL of the concentrated bulk water and biofilm suspension was analyzed for CFU, as described in Section 4.4, and the remaining volume was centrifuged at high speed (13,000 rcf, room temperature, 10 min; Eppendorf, Foster City, CA, USA). Pellets were resuspended in 200 μL of dfH_2_O and placed in a Lysing Matrix A tube (MP Biomedicals, Solon, OH, USA) along with the washed filter or biofilm swab for nucleic acid extraction as described below. 

### 4.3. Water Quality Analysis

Bulk water samples were analyzed for pH, turbidity, temperature, disinfectant residual, and heterotrophic plate count (HPC). Free chlorine and total chlorine measurements were performed using the DPD colorimetric method (Powder Pillows; Hach USA) and monochloramine and free ammonia measurements were performed using the indophenol method (method 10200, Powder Pillows, free ammonia chlorinating solution; Hach USA). HPCs were enumerated by the spread plate method on Reasoner’s 2A agar (R2A, Difco Laboratories, Detroit, MI, USA) following incubation at 28 °C for 7 d. The limit of detection (LOD) for bulk water samples was 1.0 log_10_ CFU 100 mL^−1^ and 0.7 log_10_ CFU cm^−2^ for biofilm samples. 

### 4.4. Legionella Enumeration and Presumptive Colony Analysis

For colony forming unit (CFU) enumeration, undiluted and serially diluted suspensions were spread plated on buffered charcoal yeast extract (BCYE) agar plates (BD Diagnostics, Franklin Lakes, NJ, USA) and incubated for 4–6 days at 37 °C [44]. Presumptive *Legionella* colonies were counted; and a subset was isolated and confirmed as *Legionella* spp. or *L. pneumophila* via polymerase chain reaction (PCR) using the 16S rRNA gene assays described in Section 4.6. An aliquot of the processed bulk water and biofilm samples was also heat-treated (incubation in a 55 °C water bath for 30 min) before plating on BCYE agar plates to evaluate potential differences in *Legionella* recovery from this pretreatment method [44]. Although growth of non-*Legionella* bacteria was inhibited by heat treatment, there were no significant differences between *Legionella* CFU observed between unheated and heated samples (data not shown). 

For most probable number (MPN) enumeration, Legiolert^®^ (Idexx Laboratories, Westbrook, ME, USA) was used to analyze 10 mL of the unconcentrated bulk water samples and 0.5 mL of the resuspended biofilm samples for only the Su2018, F2018, and W2019 time points. To obtain pure isolates from the Legiolert^®^ tray, positive wells were punctured using a 26-gauge needle and 50–1000 μL of the well contents was collected. A 20 μL aliquot of the sampled well was streaked onto a BCYE agar plate and incubated for 4–6 days at 37 °C. 

Those identified as *L. pneumophila* by PCR were serotyped using the Oxoid^TM^ *Legionella* Latex Agglutination Kit (ThermoFisher, Waltham, MA, USA), which allows for the separate identification of *L. pneumophila* serogroup 1 and serogroups 2–14 and detection of seven other *Legionella* species (*L. anisa*; *L. bozemanii* 1 and 2; *L. dumoffii*; *L. gormanii*; *L. jordanis*; *L. longbeachae* 1 and 2; and *L. micdadei*). Two *L. pneumophila* isolates identified as belonging to serogroups 2–14 via latex agglutination (Su2018 PVC-FC 1 and W2019 PVC-Loop Legiolert^®^ 2) and one *Legionella* spp. PCR positive isolate (F2018 PVC-FC) were sent to an external laboratory (EMSL Analytical Inc., Cinnaminson, NJ, USA) for further identification via indirect immunofluorescent antibody assay [44]. 

To account for zero values, 1 was added to all data points before conversion to the log10 scale (e.g., log10 (CFU + 1)). Calculations from CFU and molecular analyses were adjusted and expressed as units per mL or cm^2^ for bulk water samples and biofilms, respectively. The LOD for bulk water samples was 1.0 log_10_ CFU 100 mL^−1^ and 0.7 log_10_ CFU cm^−2^ for biofilm samples.

### 4.5. Isolation and Preparation of Total DNA

DNA was extracted from bacterial cells using the MasterPure™ Complete DNA purification kit (Epicentre Biotechnologies Inc., Madison, WI, USA) according to manufacturer’s protocol and the Mini-Beadbeater−16 (Biospec Products, Bartlesville, OK, USA) where samples were processed twice for 30 s at 3450 oscillations min^−1^. The DNA pellet was resuspended in 100 μL of molecular grade water.

### 4.6. Quantitative Polymerase Chain Reaction (qPCR)

Biofilm and bulk water DNA samples were analyzed in duplicate using the Applied Biosystems QuantStudio 6 Flex Fast Real-Time PCR system (ThermoFisher, Waltham, MA, USA). A 10-fold dilution of each sample was also analyzed in duplicate to test for presence of environmental qPCR inhibitors. The TaqMan qPCR assay for *Legionella* spp., *L. pneumophila*, *Mycobacterium intracellulare* detection, targeting the 16S rRNA gene, was performed as previously described [63,89,90]. The TaqMan qPCR assay for *Acanthamoeba* spp. and SYBR green qPCR assay for *Vermamoeba vermiformis* detection, targeting the 18S rRNA gene, was performed as previously described [91,92]. 

The forward and reverse primers and probe sequences (5’ to 3’) and cycling parameters used in this study for the *Legionella* spp. qPCR assay, respectively, are 16S-LegF1c: TAG TGG AAT TTC CGG TGT A; 16S-LegR1c: CCA ACA GCT AGT TGA CAT C; 16S-LegP1: CGG CTA CCT GGC CTA ATA CTG A; and 50 °C for 2 min, 95 °C for 10 min, 40 cycles of 95 °C for 10 s and 50 °C for 30 s, and at 70 °C for 30 s [90]. The forward and reverse primers and probe sequences (5’ to 3’) and cycling parameters used in this study for the *L. pneumophila* qPCR assay, respectively, are LpneuF1: CGG AAT TAC TGG GCG TAA AGG-3; LpneuR1: GAG TCA ACC AGT ATT ATC TGA CCG T; LpneuP1: AAG CCC AGG AAT TTC ACA GAT AAC TTA ATC AAC CA; and 95 °C for 10 min, 40 cycles of 95 °C for 10 s, and at 60 °C for 1 min [63]. The forward and reverse primers and probe sequences (5’ to 3’) and cycling parameters used in this study for the *M. intracellulare* qPCR assay, respectively, are F: GGG TGA GTA ACA CGT GTG CAA; R: CCA CCT AAA GAC ATG CGA CTA AA; P: TGC ACT TCG GGA TAA GCC TGG GAA A; and 50 °C for 2 min, 95 °C for 10 min, 40 cycles of 95 °C for 15 s, and 60 °C for 1 min [89]. The forward and reverse primers and probe sequences (5’ to 3’) and cycling parameters used in this study for the *Acanthamoeba* spp. qPCR assay, respectively, are TaqAcF1: CGA CCA GCG ATT AGG AGA CG; TaqAcR1: CCG ACG CCA AGG ACG AC; TaqAcP1: TGA ATA CAA AAC ACC ACC ATC GGC GC; and 50 °C for 2 min, 95 °C for 10 min, followed by 40 cycles at 95 °C for 15 s and 60 °C for 1 min, respectively [92]. The forward and reverse primer sequences (5’ to 3’) and cycling parameters used in this study for the *V. vermiformis* spp. qPCR assay, respectively, are Hv1227F: TTA CGA GGT CAG GAC ACT GT; Hv1728R: GAC CAT CCG GAG TTC TCG; and 95 °C for 3 min, followed by 40 cycles at 95 °C for 20 s, 56 °C for 30 s, and 72 °C for 40 s, and then 72 °C for 10 min [91].

For *Legionella* spp. and *L. pneumophila* qPCR assays, standard curves were generated, on each plate, using a plasmid vector (pUCIDT-AMP; Integrated DNA Technologies, Inc., Coralville, IA, USA) containing a cloned 189-bp region of the *L. pneumophila* Philadelphia-1 16S rRNA gene (NCBI reference sequence NC_002942.5, positions 609325 to 609513) that contains the targets for each of these qPCR assays. *M. intracellulare* standard curves were generated from serially diluted purified genomic DNA. Cell-based calibration curves were constructed for *Acanthamoeba* spp. and *V. vermiformis* by preparing 10-fold serial dilutions of DNA extracted from amoeba cell cultures of known densities.

Standards ranging from 1 to 10*^7^* gene copy (GC) for *Legionella* spp. and *L. pneumophila* qPCR assays; 4 to 10^4^ GC for *M. intracellulare* qPCR assays; and 1 to 10^5^ cell equivalents (CE) for the amoeba qPCR assays were generated and analyzed in triplicate along with duplicate no-template control for each 96-well plate. Data were expressed as log_10_ gene copy or CE or GU per mL or cm^2^. The limits of detection for bulk water and biofilm samples were 1.6 log_10_ GC L^−1^ and 1.3 log_10_ GC cm^−2^ for the *Legionella* spp. and *L. pneumophila* assays; 1.3 log_10_ GC L^−1^ and 0.9 log_10_ GC cm^−2^ for the *M. intracellulare* assay; 1.4 log_10_ CE L^−1^ and 1.0 log_10_ CE cm^−2^ for the *Acanthamoeba* spp. assay; and 2.4 log_10_ CE L^−1^ and 2.0 log_10_ CE cm^−2^ for the *V. vermiformis* assay, respectively.

### 4.7. Whole Genome Sequencing and Sequence Analyses

Twenty-one bulk water isolates were chosen for whole genome sequencing. Total DNA from each strain was isolated as described in Section 4.5. DNA concentrations were estimated using the Nanodrop ND−1000 Spectrophotometer (NanoDrop Technologies, Inc., Wilmington, DE, USA). Total DNA was submitted for whole genome sequencing (Wright Labs LLC, Huntingdon, PA, USA) where genomic libraries were prepared using the Nextera XT Index Kit v2 Set A and sequenced on the HiSeq 4000 platform (Illumina Inc., San Diego, CA, USA) with a HiSeq 3000/4000 PE Cluster kit (2 × 150 bp). Prior to assembly, libraries were (i) cleaned from contaminants (adapters, phiX, artifacts, and human), (ii) error corrected, (iii) normalized to ≤ 100 ×, (iv) removed of low (< 6 ×) coverage reads, and (v) filtered to a minimum length read of 100 nt. Reads were processed using the software package BBMap v37.90 (http://sourceforge.net/projects/bbmap) and de novo assembly using the software Unicycler v0.4.4 [93]. The Illumina reads are deposited in the National Center for Biotechnology Information (NCBI) Sequence Read Archive database under the BioProject accession number PRJNA558750.

Sequence-based typing (SBT) analysis was performed in silico with legsta and multi-locus sequence typing (mlst) as described previously [94]. The phylogenetic tree was constructed combining the sequenced genomes from this study and a set of closely related genomes. Relatedness is determined by alignment similarity to a select subset of COG (Clusters of Orthologous Groups) domains. The phylogenetic tree is reconstructed using FastTree 2 [95] to determine maximum likelihood phylogeny. Average nucleotide identity (ANI), an index of similarity between two genomes [96], was calculated using FastANI v1.3 (https://github.com/ParBLiSS/FastANI) [97]. ANI is defined as mean nucleotide identity of orthologous gene pairs shared between two microbial genomes. No ANI output is reported for a genome pair if the ANI value is below 80%.

### 4.8. Statistical Analysis

For each water quality parameter, a Shapiro–Wilk normality test was conducted for each site to determine distribution of the data throughout the sampling period. A one-way analysis of variance (ANOVA) using the Tukey multiple comparisons test was conducted between each site and sample type. *P*-values of < 0.05 were considered statistically significant. Analyses were performed using Prism 8 (GraphPad Software, San Diego, CA, USA). The R functions cor() and cor.test() were used, with a Spearman correction, to determine the direction (positive or negative) and significance of correlation between pairs of water quality characteristics within each sample location [98]. The correlation between the pairs was denoted by the number, r, which varies between –1 and +1, with 0 meaning no correlation, +1 a complete positive correlation, and –1 a complete negative correlation.

## Figures and Tables

**Table 1:** Description of each sampling location.

	Sampling Locations											
Site Name	PVC-MA		PVC-R		PVC-FC		Spigot		Faucet		Fountain	
**Disinfectant type**	monochloramine		chlorine		chlorine		chlorine		chlorine		chlorine	
**Water type**	potable water		chiller water		potable water		potable water		potable water		potable water	
**Approx. usage per season**	172,709 L		1,492,209 L		5 L		1703 L		864 L		689 L	
**Outlet type**	valve		valve		valve		spigot		faucet		bubbler	
**Outlet material**	PVC80; PharMed BPT^1^		PVC80		PVC80		cast brass		chrome plated, forged brass		SS/EDPM rubber	
**Pipe material**	PVC80		FDI and PPT^2^		PVC80 and copper^2^		copper		copper		copper	
**Total distance to feed water**	14 m	46 ft	396 m	1299 ft	21 m	70 ft	102 m	334 ft	20 m	65 ft	9.7 m	32 ft
Section 1	14 m	46 ft	366 m	1201 ft	9 m	30 ft	5 m	16 ft	8 m	26 ft	9.1 m	30 ft
**pipe diameter**	15 cm	6 in	7.6 cm	3 in	2.5 cm	1 in	1.9 cm	0.75 in	1.9 cm	0.75 in	1.3 cm	0.5 in
Section 2	-		30 m	98 ft	12 m	40 ft	97 m	318 ft	12 m	39 ft	0.6 m	2 ft
**pipe diameter**	-		1.3 cm	0.5 in	2.5 cm	1 in	5.1 cm	2 in	5.1 cm	2 in	0.6 cm	0.25 in

Abbreviations: -, not applicable; cm, centimeter; EDPM, ethylene propylene diene; FDI, fiberglass ductile iron; ft, feet; in, inch; m, meter; PPT, PVC-reinforced plastic tubing; PVC80, polyvinyl chloride schedule 80; SS, stainless steel. ^1^ Masterflex PharMed BPT tubing approximately 1 m (3.5 ft) in length and inner diameter 4.8 mm (0.189 in). ^2^ Section 1 and Section 2, respectively.

**Table 2:** Summary of water quality parameters for each sampling location.

Location Name & Sample Type		HPC		pH		Turbidity		Temp		Free Cl_2_		Total Cl_2_	
		Mean	SEM	Mean	SEM	Mean	SEM	Mean	SEM	Mean	SEM	Mean	SEM
PVC-MA ^a^	First Draw	5.5	0.1	8.21	0.08	0.23	0.03	20.2	0.3	0.06	0.01	1.21	0.11
	Second Draw	4.9	0.2	8.19	0.07	0.26	0.04	20.2	0.3	0.06	0.01	1.22	0.11
	Biofilm	3.4	0.2	-	-	-	-	-	-	-	-	-	-
PVC-R	First Draw	5.4	0.2	8.53	0.07	6.94	3.23	24.8	1.4	1.04	0.10	1.13	0.09
	Second Draw	4.5	0.2	8.68	0.05	2.13	0.96	24.8	1.4	0.90	0.06	1.02	0.06
	Biofilm	2.6	0.1	-	-	-	-	-	-	-	-	-	-
PVC-FC	First Draw	4.2	0.6	8.47	0.15	0.42	0.15	20.7	2.5	0.83	0.07	0.92	0.08
	Second Draw	4.0	0.1	8.60	0.17	0.22	0.02	18.6	3.0	0.99	0.04	1.10	0.05
	Biofilm	2.0	0.6	-	-	-	-	-	-	-	-	-	-
Spigot	First Draw	5.4	0.1	8.67	0.06	0.81	0.35	24.8	1.2	0.53	0.12	0.59	0.12
	Second Draw	2.8	0.2	8.73	0.06	0.23	0.08	18.4	2.3	1.00	0.06	1.12	0.04
	Biofilm	2.5	0.2	-	-	-	-	-	-	-	-	-	-
Faucet	First Draw	5.5	0.2	8.67	0.06	0.17	0.02	24.0	0.7	0.35	0.10	0.40	0.11
	Second Draw	4.2	0.2	8.72	0.06	0.18	0.02	20.0	1.7	1.05	0.04	1.13	0.04
	Biofilm	4.2	0.2	-	-	-	-	-	-	-	-	-	-
Fountain	First Draw	4.0	0.2	8.75	0.06	0.25	0.06	13.5	0.4	0.59	0.07	0.69	0.07
	Second Draw	3.8	0.3	8.78	0.07	0.18	0.02	15.4	1.0	0.89	0.03	0.98	0.03
	Biofilm	4.0	0.5	-	-	-	-	-	-	-	-	-	-

Abbreviations: -, no data; Cl_2_, chlorine, mg L^−1^; HPC, heterotrophic plate count, log_10_ CFU per 100 mL or cm^2^; LOD, limit of detection; NTU, Nephelometric Turbidity Unit; SEM, standard error mean; Temp, temperature in degrees Celsius. ^a^ see Materials and Methods, Section 4.1, for average monochloramine and free ammonia levels during the entire sampling period.

**Table 3:** Summary of *Legionella* spp. culture results for positive sites.

Time Point	PVC-R			PVC-FC						Faucet								
	First Draw			First Draw			Second Draw			First Draw			Second Draw			Biofilm		
	CFU	sg	PCR	CFU	sg	PCR	CFU	sg	PCR	CFU	sg	PCR	CFU	sg	PCR	CFU	sg	PCR
F2016	-	-	-	nd	nd	nd	nd	nd	nd	-	-	-	-	-	-	1.9	1 & 2-14	Leg/Lp
W2017	-	-	-	nd	nd	nd	nd	nd	nd	-	-	-	-	-	-	-	-	-
Sp2017	-	-	-	nd	nd	nd	nd	nd	nd	1.2	2-14	Leg/Lp	-	-	-	-	-	-
Su2017	-	-	-	-	-	-	-	-	-	-	-	-	-	-	-	-	-	-
F2017	-	-	-	-	-	-	-	-	-	1.2	neg	Leg	-	-	-	-	-	-
W2018	2.6	neg	Leg	4.5	neg	Leg	2.6	neg	Leg	2.1	1 & 2-14	Leg/Lp	-	-	-	-	-	-
Sp2018	-	-	-	-	-		-	-	-	-	-	-	-	-	-	-	-	-
Su2018	-	-	-	3.8	neg & 2-14	Leg & Leg/Lp	2.8	neg & 2-14	Leg & Leg/Lp	2.7	1	Leg/Lp	-	-	-	-	-	-
F2018	-	-	-	-	-		3.0	neg	Leg	-	-	-	-	-	-	-	-	-
W2019	-	-	-	2.1	neg	Leg	2.6	neg	Leg	2.8	1	Leg/Lp	2.1	1	Leg/Lp	-	-	-

No culturable *Legionella* was detected in the PVC-MA; Spigot; Fountain; PVC-R Distal and Biofilm; and PVC-FC Biofilm samples. Abbreviations: -, below LOD; B, biofilm; LOD, limit of detection; nd, no data; qPCR, quantitative PCR; sg, serogroup. CFU is expressed as log_10_ CFU 100 mL^−1^ for bulk water samples (LOD 1.0 log_10_ CFU 100 mL^−1^) and log_10_ CFU cm^−2^ for biofilm samples (LOD 0.7 log_10_ CFU cm^−2^). sg results: 1, sg 1 positive; 2-14, sg 2-14 positive; 1 & 2-14, different colonies on the plate were sg 1 and 2-14 positive; neg, agglutination negative. PCR results: Leg, *Legionella* spp. assay positive only; Leg/Lp, *Legionella* spp. and *L. pneumophila* assay positive.

**Table 4:** Summary statistics of whole-genome assemblies for the drinking water isolates

Isolate	Lineage	Genome Size (bp)	No. of Contigs	Contig *N*_50_ (bp)	G+C Content (%)	No. Predicted Genes	MLST
Su2018 Faucet 1	*L. pneumophila*	3,589,286	72	160,018	38	3240	1
Su2018 Faucet 2	*L. pneumophila*	3,589,059	75	160,010	38	3239	1
Su2018 Faucet 3	*L. pneumophila*	3,589,228	71	160,018	38	3236	1
Su2018 Faucet 4	*L. pneumophila*	3,574,346	99	88,934	38	3228	1
Su2018 Faucet 5	*L. pneumophila*	3,590,303	69	160,010	38	3240	1
W2019 Faucet Legiolert® 1	*L. pneumophila*	3,564,531	124	59,538	38	3229	1
W2019 Faucet 2	*L. pneumophila*	3,562,418	77	123,108	38	3224	1
Su2018 PVC-FC 1	*L. pneumophila*	3,510,698	39	413,452	38	3151	2037
Su2018 PVC-FC 2	*L. pneumophila*	3,498,273	40	223,695	38	3138	2037
Su2018 PVC-FC 3	*Legionella*	3,285,021	57	260,915	41	3028	-
F2018 PVC-FC	*Legionella*	3,287,868	49	348,799	41	3028	-
W2019 PVC-FC 1	*Legionella*	3,063,339	43	348,799	41	2812	-
W2019 PVC-FC 2	*Legionella*	3,288,042	53	321,108	41	3033	-
W2019 PVC-FC 3	*Legionella*	3,286,784	53	283,321	41	3031	-
W2019 PVC-FC 4	*Legionella*	3,285,778	45	466,387	41	3022	-
W2019 PVC-Loop Legiolert® 1	*Ochrobactrum*	4,764,477	55	478,728	58	4621	-
W2019 PVC-Loop Legiolert® 2	*L. pneumophila*	3,388,353	42	255,126	38	3057	1950
W2019 BWS Supply Line 1	*L. pneumophila*	3,589,200	67	176,930	38	3237	1
W2019 BWS Supply Line 2	*L. pneumophila*	3,588,658	70	160,010	38	3242	1

Abbreviations: -, not applicable; bp, base pair; BWS, building water system; LST, multi-locus sequence typing; No., number.

## Supplementary Materials

The following are available online at https://www.mdpi.com/2076-0817/9/7/567/s1; Figure S1: Images of outlets at each sampling location; Figure S2: Scatterplot of turbidity measurements for bulk water samples; Figure S3: Scatterplot of free (A) and total (B) chlorine measurements for bulk water samples.
