# Supplementary material for: Legionella Diversity and Spatiotemporal Variation in the Occurrence of Opportunistic Pathogens within a Large Building Water System
Source: Pathogens. 2020 Jul 13;9(7):567. doi: 10.3390/pathogens9070567 (PMC7400177; doi:10.3390/pathogens9070567)
Supplement: Supplementary file 1 [file pathogens-09-00567-s001.pdf]

pathogens

Supplementary Materials

Legionella Diversity and Spatiotemporal Variation in the Occurrence of Opportunistic Pathogens within a Large Building Water System

Helen Y. Buse [1,*], Brian J. Morris [2], Vicente Gomez-Alvarez [3], Jeffrey G. Szabo [1], and John S. Hall [1]

1 Homeland Security and Materials Management Division, Center for Environmental Solutions & Emergency Response (CESER), Office of Research and Development (ORD), US Environmental Protection Agency (USEPA), Cincinnati, OH 45268, USA
2 Pegasus Technical Services, Inc c/o US EPA, Cincinnati, OH 45268, USA
3 Water Infrastructure Division, Center for Environmental Solutions & Emergency Response (CESER), US Environmental Protection Agency (USEPA), Office of Research and Development (ORD), Cincinnati, OH 45268, USA
* Correspondence: buse.helen@epa.gov; Tel.: +1-513-569-7930

Received: 8 June 2020; Accepted: 7 July 2020; Published: date

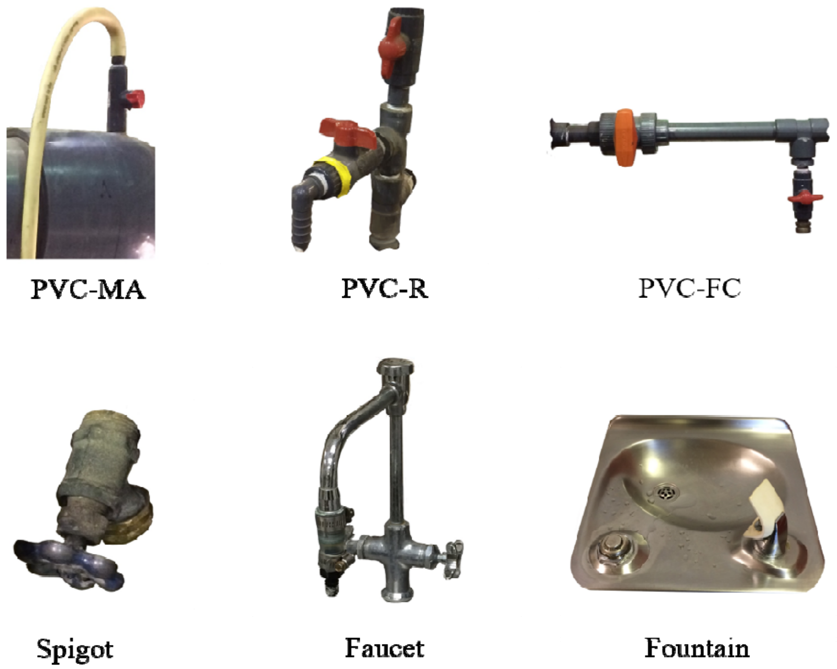

Figure S1. Images of sampling outlets at each location

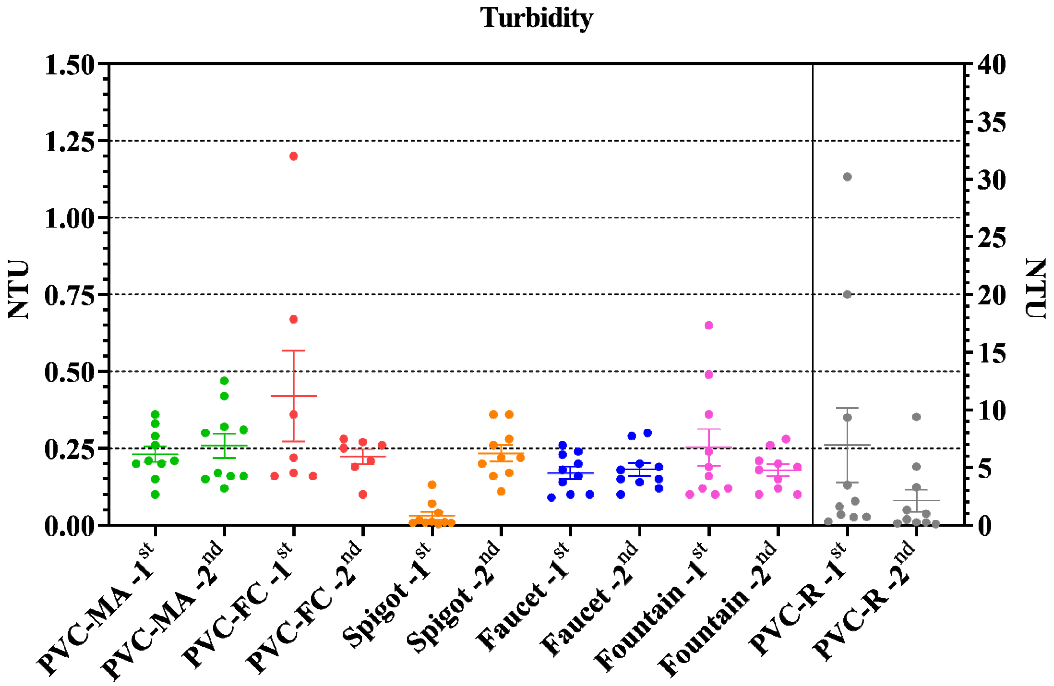

Figure S2. Scatterplot of turbidity measurements for bulk water.

Mean and standard error mean lines are shown for turbidity measurements at each site for all sampling time points. PVC-R -1st and -2nd samples are plotted on the right y-axis. All other samples are plotted on the left y-axis.

(a)

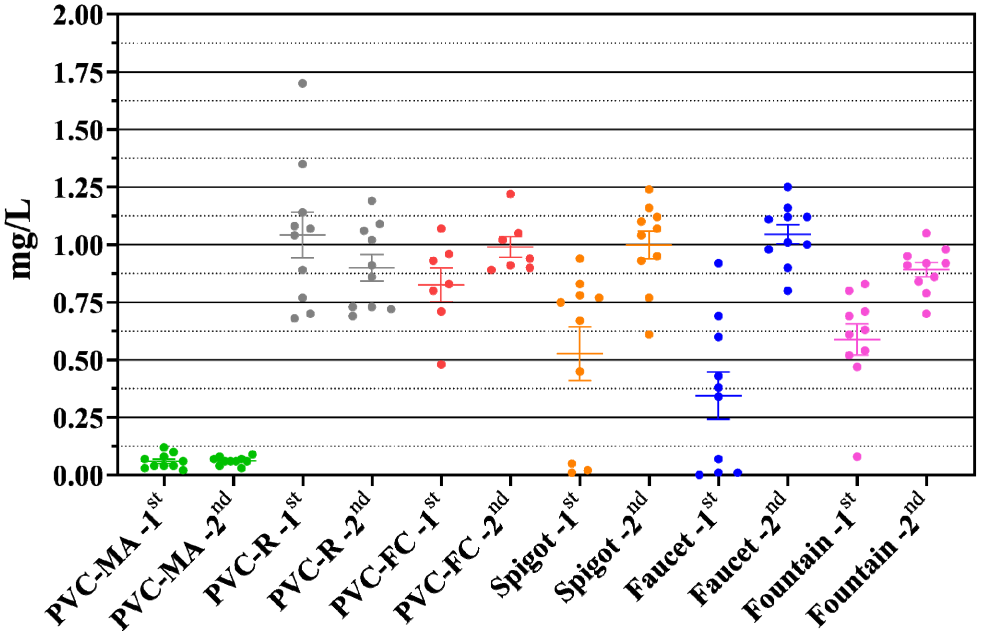

(b)

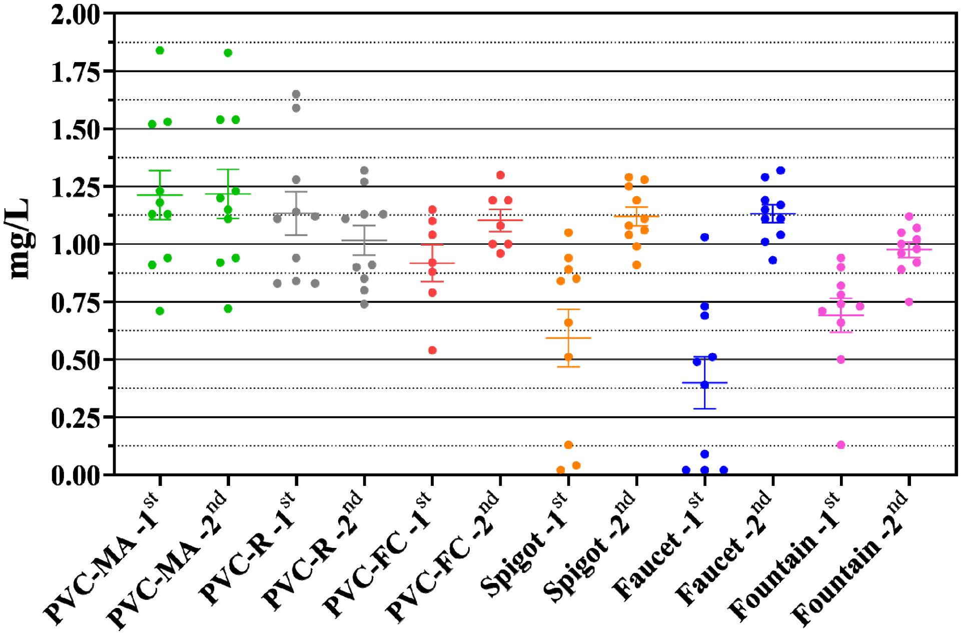

Figure S3. Scatterplot of chlorine measurements for bulk water

Mean and standard error mean are shown for free (a) and total (b) chlorine measurements at each site for all sampling time points. 1st, first draw bulk water samples. 2nd, second draw/post-flushing bulk water samples.

© 2020 by the authors. Submitted for possible open access publication under the terms and conditions of the Creative Commons Attribution (CC BY) license (http://creativecommons.org/licenses/by/4.0/).
